# Extranodal NK-/T-cell lymphoma, nasal type: what advances have been made in the last decade?

**DOI:** 10.3389/fonc.2023.1175545

**Published:** 2023-07-17

**Authors:** Renata de Oliveira Costa, Juliana Pereira, Luís Alberto de Pádua Covas Lage, Otávio César Guimarães Baiocchi

**Affiliations:** ^1^ Department of Hematology, Faculdade de Ciências Médicas de Santos (FCMS), Centro Universitário Lusíadas (Unilus), Santos, São Paulo, Brazil; ^2^ Hospital Alemao Osvaldo Cruz (HAOC), São Paulo, Brazil; ^3^ Department of Hematology, Hemotherapy and Cell Therapy, Faculdade de Medicina da Universidade de Sao Paulo (FM-USP), São Paulo, Brazil; ^4^ Laboratory of Medical Investigation in Pathogenesis and Directed Therapy in Onco-Immuno-Hematology (LIM-31), University of Sao Paulo (USP), São Paulo, Brazil; ^5^ Department of Hematology, Universidade Federal de Sao Paulo (Unifesp), São Paulo, Brazil

**Keywords:** extranodal NK-/T-cell lymphoma, nasal type (ENKTCL-NT), Epstein-Barr virus (EBV), angiocentricity, JAK/STAT pathway, extended-field radiotherapy (EF-RT), multi-drug resistance (MDR), prognosis

## Abstract

Extranodal NK-/T-cell lymphoma (ENKTCL) is a rare and highly aggressive malignancy with significant racial and geographic variations worldwide. In addition to the formerly “nasal-type” initial description, these lymphomas are predominantly extranodal in origin and typically cause vascular damage and tissue destruction, and although not fully understood, Epstein–Barr virus (EBV) has an important role in its pathogenesis. Initial assessment must include a hematopathology review of representative and viable tumor areas without necrosis for adequate immunohistochemistry studies, including EBV-encoded small RNA (EBER) *in situ* hybridization (ISH). Positron emission tomography with 18-fluorodeoxyglucose (^18^F-FDG-PET/CT) for accurate staging is essential, and most patients will have localized disease (IE/IIE) at diagnosis. Apart from other T-cell malignancies, the best treatment even for localized cases is combined modality therapy (chemotherapy plus radiotherapy) with non-anthracycline-based regimens. For advanced-stage disease, l-asparaginase-containing regimens have shown improved survival, but relapsed and refractory cases have very poor outcomes. Nowadays, even with a better understanding of pathogenic pathways, up-front therapy is completely based on chemotherapy and radiotherapy, and treatment-related mortality is not low. Future strategies targeting signaling pathways and immunotherapy are evolving, but we need to better identify those patients with dismal outcomes in a pre-emptive way. Given the rarity of the disease, international collaborations are urgently needed, and clinical trials are the way to change the future.

## 1 Introduction

Peripheral T- and NK-cell lymphomas (PTCLs) are rare, heterogeneous, and commonly aggressive non-Hodgkin’s lymphomas (NHLs) that originate from post-thymic T-lymphocytes and NK cells. Altogether, these disorders comprise 10%–15% of all lymphoma subtypes with great geographic differences (1). Although it has been 50 years since the recognition that NHLs are derived from either B or T cells, T- and NK-cell lymphomas are still poorly understood malignancies due to their low incidence when compared to B-cell lymphomas. In the past, lethal midline granuloma or midline malignant reticulosis were terms used to describe the extranodal NK-/T-cell lymphoma, nasal-type (ENKTCL-NT), a lymphoma subtype that typically involves the midline facial structures. Nowadays, a much better understanding regarding clinical behavior has led to the qualifier topography “nasal-type” drop off in the present fifth edition of the World Health Organization Classification of Hematolymphoid Malignancies, as many extranodal non-nasal cases were well recognized, and dismal outcomes regarding topography need yet to be addressed (2, 3).

The ENKTCL diagnosis is based on clinical aspects, in addition to histopathologic features, expression of standard cytotoxic molecules and CD56 (neural cell adhesion molecule (NCAM)]. EBV is usually present and corroborate an accurate diagnosis. More common in Asian, Central, and South American citizens, Epstein–Barr virus (EBV) DNA and its oncogenic proteins are present in virtually all cases of ENKTCL. Fortunately, the pathogenesis of this disease has been extensively studied, and the identification of different oncogenic intracellular signaling pathways, such as Janus kinase/signal transducer and activator of transcription (JAK/STAT), programmed cell death-1/programmed death ligand 1 (PD-1/PD-L1), and epigenetic dysregulation, brought new insights to better translate these biological advances into clinical practice (4, 5).

After appropriate staging with functional images, ENKTCL must be classified into localized or advanced-stage disease. Being one of the most radiosensitive NHLs, the disease stage is the most important factor to define treatment proposals and to predict survival. Prognostic parameters, such as age, disease stage, and EBV load, may be used, but predictive models are not sufficiently accurate to guide treatment. In addition to the crucial role of radiotherapy, especially for early-stage ENKTCL, it is now known that conventional anthracycline-based chemotherapy is not sufficient. Concurrent or sequential chemoradiotherapy (CCRT or SCRT) is the standard of care for localized disease, and l-asparaginase based-chemotherapy should be used for either localized or advanced stage. In this review, we summarize current practical approaches to disease staging, available treatment options, and new insights that can guide us to better future outcomes.

## 2 Epidemiology and clinical features

ENKTCL is an uncommon and predominantly extranodal malignancy with great racial and geographic diversity. In the *International T-cell Lymphoma Project* (ITCLP), with the participation of 22 institutions from North America, Asia, and Europe, ENKTCL was found to be responsible for 2.7% of all cases (6). When analyzed by geographic region, the differences became evident with ENKTCL corresponding to 5.1% cases in North America, 4.3% in Europe, and impressive 22.4% cases in Asia. Another population-based registry comparing hematological malignancies from Japan and the USA demonstrated an age-adjusted incidence rate of ENKTCL of 0.04 in the USA and 0.1 per 100,000 inhabitants in Japan (7). In addition, Latin America seems to be another geographic area with a high prevalence of ENKTCL, particularly in countries such as Guatemala and Brazil (8, 9). An interesting study comparing Mexican patients with other Latin American cases demonstrated that ENKTCL was the most frequent NK-/T-cell lymphoma, representing 40% of all these cases. Interestingly, Mexico is a country geographically located in North America (10). Possibly ethnic susceptibility may partly explain this pattern, as the Mongoloid race from Asia is genetically related to natives from Central and South America (11). Of note, a familial occurrence of ENKTCL affecting father and son 2 years apart was documented. Both were farmers and used large amounts of pesticides (12). Occupation, especially organophosphate exposure, may also play a potential role in its lymphomagenesis (13).

**Figure 1:**
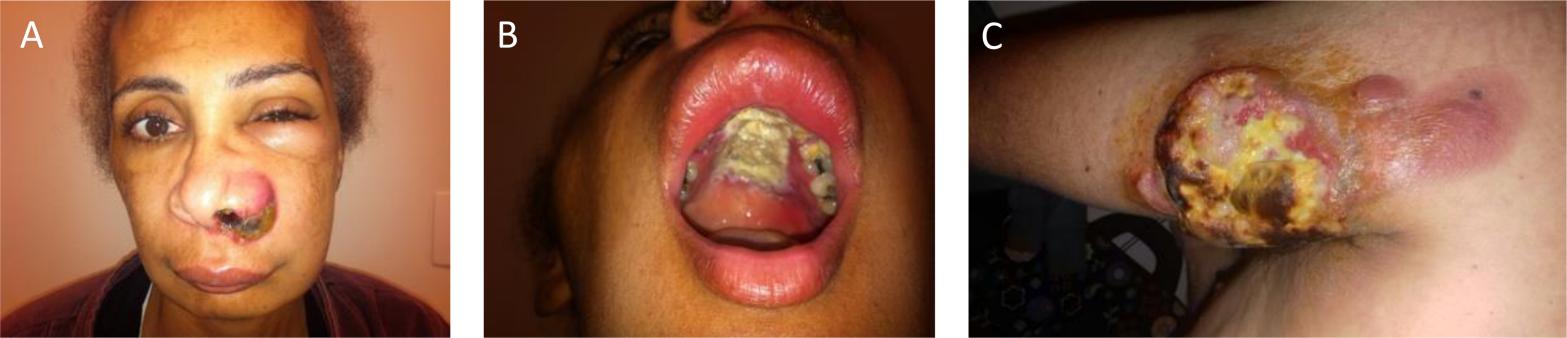
Clinical presentations of ENKTCL. **(A)** Classical nasal type with extensive and destructive lesions and eyelid swelling. **(B)** Same patient with perforation of hard palate. **(C)** Another patient with primary bulky cutaneous presentation. ENKTCL, extranodal NK-/T-cell lymphoma.

ENKTCL has a slight male predominance with a median age of 50 years at diagnosis. The most common initial symptom is nasal obstruction and discharge, explained by the localized upper aerodigestive tract presentation in most patients (14). Locally aggressive, it often causes hard palate perforation and destroys midfacial structures, such as paranasal sinuses and nasopharynx, leading to cartilage and bone destruction, with great local deformity (**Figures 1A, B**). Likewise, it may extend to contiguous tissues, such as the orbit or eyelid, and although not common, cranial nerves and meninges may be affected (15, 16). Systemic disease is highly aggressive but may occur, and the most common affected sites include the skin (**Figure 1C**), soft tissue, testis, and gut (17, 18). Extranodal primary disease may occur even without nasal involvement, which led to the recent nomenclature change in the upcoming 5th Edition of the World Health Organization Classification of Hematolymphoid Malignancies (3). In a Brazilian retrospective cohort analysis, almost 20% of cases were extranasal in origin, and, as bone marrow involvement is rare, pancytopenia should draw attention to the diagnosis of hemophagocytic syndrome. Systemic symptoms are variable and may occur, especially in advanced disease (19). Primary extranasal disease seems to confer worse outcomes, even when compared to stage III/IV nasal disease (18).

## 3 Diagnosis: cell of origin, pathology, and immunophenotype

Early diagnosis of ENKTCL may be challenging if extensive tumor necrosis caused by blood vessel infiltration by neoplastic cells exists (angiocentricity) and often requires multiple biopsies. Prompt clinical diagnosis is rare since most patients can be misdiagnosed and treated for more common clinical conditions, such as acute rhinosinusitis. Differential diagnoses are broad and may include cutaneous leishmaniasis, Wegener granulomatosis, South American blastomycosis, tuberculosis, leprosy, EBV-positive mucocutaneous ulcer, nasopharyngeal carcinoma, and lymphomatoid granulomatosis (20, 21). In addition, outside endemic areas and given the NK-/T-cell nature of the neoplasm, a hematopathologist is usually required for adequate diagnosis (22). ENKTCL mainly originates in activated NK cells, lacking T-cell receptor (TCR) genes, and less commonly, the cell of origin (COO) may be a cytotoxic T cell with rearranged TCR genes (23). There are little data in the literature regarding clinical, pathological, phenotypic, and molecular–genetic differences between ENKTCL cases derived from T- or NK-cell origin. Data regarding therapeutic response are virtually non-existent due to the COO. **Table 1** summarizes the main differences between ENKTCL according to its COO.

**Table 1:** Main differences between cases of ENKTCL according to their COO.

	ENKTCL—COO: NK cell	ENKTCL—COO: T-CD8+
Frequency ^#^	More than 75% of cases	Up to 25% of cases (αβ+, γδ+, or αβ/γδ+)
CD56 expression *	83%	33%
CXCL-13 expression *	59%	0%
PD-1 expression *	0%	40%
OCT-2 expression *	38%	0%
IRF-4/MUM-1 expression *	54%	20%
Gender predominance ^#^	Male	Equal among both genders
Prognosis (for early-stage cases—IE/IIE) ^#^	Poor	Tendency to better survival
Mutational landscape (SNV/CNV) ***	*STAT3*, *DDX3X*, *KMT2C*, *JAK2*, *KMT2D*, *EP300*, *STAT5B*, *STAT5A*	*EPHA1*, *TP53*, *ARID1A*, *PTPRQ*, *NCOR2*, *PPFIA2*, *BCOR*, *PTPRK*, *HDAC*
Up-regulated signaling pathways ***	JAK/STAT	RAS-MAPK and epigenetic modifiers
Potential therapeutic applications ***	JAK inhibitors	HDACi

ENKTCL, extranodal NK-/T-cell lymphoma; COO, cell of origin; NK, natural killer; OS, overall survival; SNV, single-nucleotide variant; CNV, copy number variation; HDACi, histone deacetylase inhibitors.

^#^According to Swerdlow SH et al. (2017) (24), Pongpruttipan et al. (2012) (23), Hong et al. (2016) (25), and Jhuang JY et al. (2015) (26).

*According to Pongpruttipan et al. (2012) (23).

***According to Xiong et al. (2020) (27).

**Figure 2:**
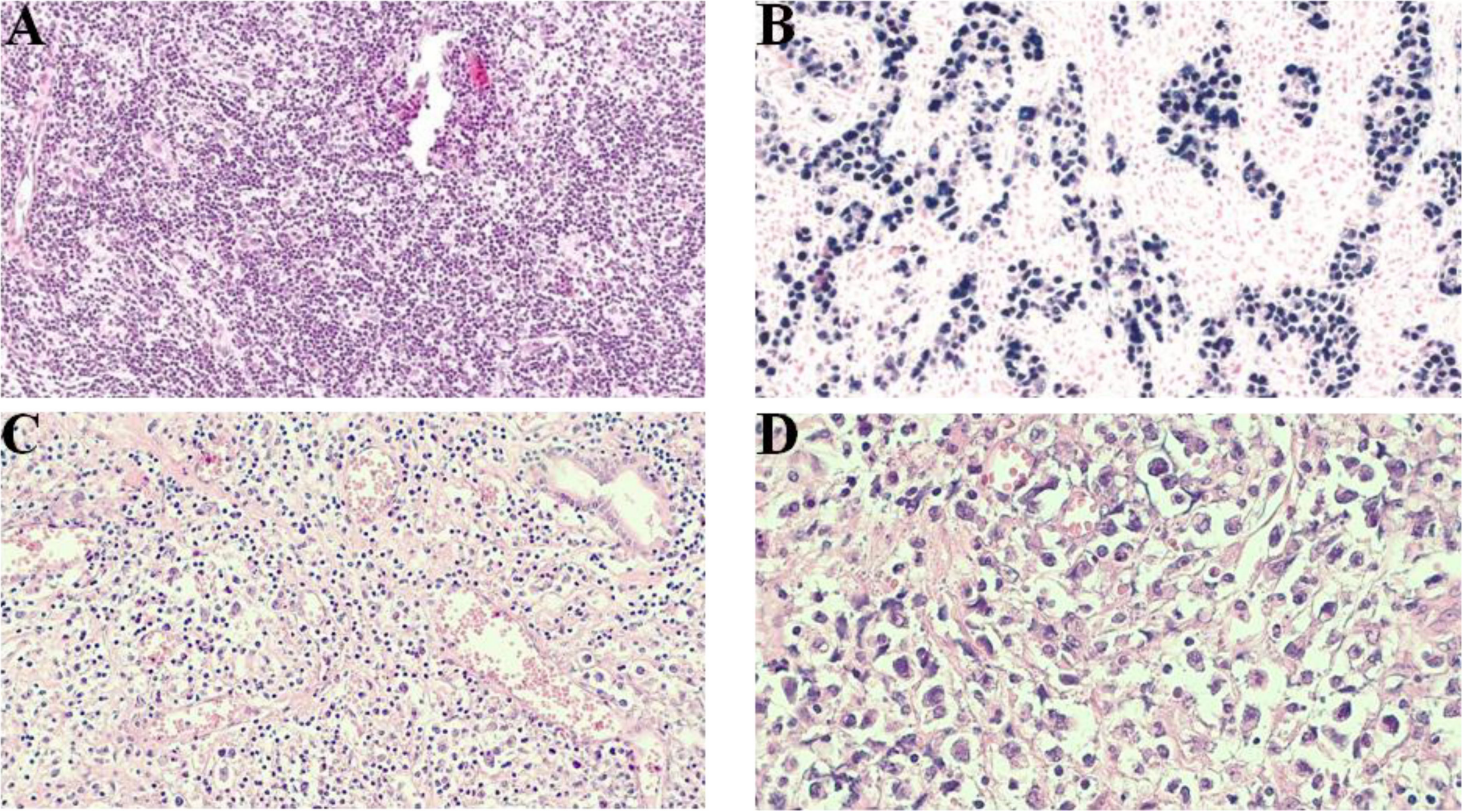
Microscopy of ENKTCL, nasal-type. **(A)** Diffuse dense tissue infiltration by small- and medium-sized atypical cells (H&E, optical microscopy, ×10 magnification). **(B)** Strong positivity for EBV-encoded small RNA by ISH for neoplastic lymphoid cells. In this staining, some large-sized atypical cells were highlighted (formalin-fixed, paraffin-embedded [FFPE] sample, optical microscopy, ×40 magnification). **(C)** Atypical lymphoid infiltration and marked vessel proliferation (optical microscopy, ×20 magnification). **(D)** High-power field demonstrating small- to medium-sized neoplastic cells and rare pleomorphic large cells infiltrating the connective tissue (optical microscopy, ×100 magnification). ENKTCL, extranodal NK-/T-cell lymphoma; EBV, Epstein–Barr virus; ISH, *in situ* hybridization.

Histologically, ENKTCL has a wide-ranging cytologic spectrum, with atypical and highly pleomorphic cellular infiltrate, with small, medium, or large and hyperchromatic cells. Most cases demonstrate medium-sized cells, admixed with small and large ones (**Figures 2A, C, D**). The presence of angioinvasion, angiodestruction, and necrosis is a hallmark of this tumor (**Figures 2A, C**). There may also coexist reactive inflammatory cells like lymphocytes, plasma cells, histiocytes, and eosinophils. Pseudoepitheliomatous hyperplasia, a reactive epithelial proliferation mimicking invasive squamous cell carcinoma, has been reported (24, 28). Immunophenotyping is very helpful in confirming the diagnosis, and malignant cells typically have an NK phenotype, with usually positivity for CD56. Surface CD3 negativity but cytoplasmic CD3+ (CD3ϵ) cells on paraffin samples help to support the diagnosis. There is variable expression of FAS, FASL, CD7, CD25, and CD30. Other NK- and T-cell antigens, such as CD57, CD16, CD4, and CD8, are usually negative, and a small subset of cases is truly T cell in origin. *In situ* hybridization (ISH) for EBV-encoded small RNA (EBER) is a reliable way to demonstrate the presence of EBV (**Figure 2B**) (23, 24, 29). Cytotoxic granules like perforin, TIA-1, and granzyme-B are usually positive. Granulysin also seems to be a useful marker for ENKTCL, especially when lacking expression of other common markers (30, 31).

## 4 Pathogenesis

### 4.1 Recurrent genomic aberrations in ENKTCL and mutational profile

The etiopathogenesis of ENKTCL is complex and not completely elucidated. However, genetic analysis using comparative genomic hybridization (CGH), array CGH (aCGH), and loss of heterozygosity (LOH) assays showed recurrent genetic alterations in ENKTCL. The most common were gains at chromosomes 1p, 6p, 11p, 12q, 17q, 20q, and Xp and losses at chromosomes 6q, 11q, 13q, and 17p (32–37). The losses at chromosome 6q are associated with the loss of tumor-suppressor genes, such as *FOXO3*, *HACE1*, *PRDM1*, *ATG5*, and *AIM1* (38, 39). The *TP53* mutation was described in 31% to 63% of ENKTCL in two Asian population cohorts (40, 41). Therefore, ENKTCL is a malignancy associated with a high degree of chromosomal instability. Siu LL et al. reported LOH on chromosomes 6q and 13q in 80% and 66.7% of the cases, respectively, when they studied 15 patients with NK-cell lymphomas (32). Likewise, a study conducted by Chen CY et al. analyzing the pattern and distribution of recurrent karyotypic abnormalities in 200 Chinese patients with NHL demonstrated an increased frequency of 1q duplication, 6p duplication, and 11q deletion in ENKTCL in comparison to other NHL subtypes (42).

Noteworthy, the tumor mutational burden in ENKTCL is remarkably lower than in other aggressive lymphomas, similar to that found in EBV-positive nasopharyngeal carcinomas and gastric carcinoma (27, 43, 44), supporting the importance of EBV in the pathogenesis of EBV-positive neoplasms. Although recurrent somatic mutations (single-nucleotide variant (SNV)) have been reported at high frequency in ENKTCL, particularly those involving the *DDX3X* RNA helicase, *TP53*, JAK/STAT pathway genes, and epigenetic modifiers, its tumor mutational burden (TMB) is usually lower than observed in other NHLs, such as the diffuse large B-cell lymphoma (DLBCL). In this sense, a recent study accessed TMB in 188 tumor samples and 98 plasma samples from patients with different subtypes of NHLs, characterizing the landscape of somatic mutations between high-TMB (TMB-H) and low-TMB (TMB-L). The cutoff value defined to characterize TMB-H was the top quartile TMB distribution. In this study, 0% of the tumor and 0% of plasma samples from ENKTCL patients were categorized as TMB-H, compared to 34.09% (tumor) and 34.25% (plasma) of samples from DLBCL patients (45). Similarly, in another study conducted by Cho J et al. using a massively parallel sequencing panel involving 405 genes in 300 patients with different NHL subtypes, the number of SNV/indel was significantly higher (p < 0.001) in patients with aggressive B-cell lymphomas compared to T-lineage and natural-killer lymphomas (46). Additionally, mutations in genes involved in the JAK/STAT pathway, epigenetic modification, RNA helicase family, RAS-MAP kinase pathway, and tumor suppressor genes contribute to ENKTCL lymphomagenesis (4, 27, 47–53). Epigenetic dysregulation involving *BCOR* and *EZH2* was also described, and its biological relevance to the ENKTCL scenario is being investigated (49, 54).

### 4.2 The role of EBV in ENKTCL oncogenesis

The EBV is a ubiquitous gamma human herpesvirus classified as a group 1 carcinogen by the *International Agency for Research on Cancer* (55). Mostly acquired in childhood in subclinical forms, more than 90% of adults have a lifelong asymptomatic latent disease. EBV presents tropism for different cells, including B-lymphocytes, natural killer cells, and T-lymphocytes (56, 57) being associated with several lymphoproliferative diseases (LDs) (**Table 2**). During primary infection, EBV usually infects epithelial cells and B-lymphocytes, but occasionally, it can infect some cells of the -T/-NK lineage. In individuals with a poor presentation by specific human leukocyte antigens (defective HLA class II molecules, such as HLA-DPB1 and HLA-DRB1) or with genetic predisposition, EBV-infected -T/-NK cells can evade host immunity and consequently survive and proliferate (63). With the participation of viral oncoproteins, such as latent membrane protein (LMP) and EBV-encoded nuclear antigens (EBNAs) and accumulation of genetic mutations, such as those affecting *DDX3X* and *TP53* genes or modifications in epigenetic targets (*KMT2C*, *KMT2D*, and *TET2*), selection and subsequent clonal expansion occur, leading to the subsequent development of ENKTCL (27, 64, 65).

**Table 2:** EBV positivity by lymphoma subtype.

Lymphoma subtype	EBV positivity (%)	Reference
Diffuse large B-cell lymphoma	≈10-15	(3, 24, 58)
Hodgkin lymphoma	≈30–40	(3, 24, 59)
Peripheral T-cell lymphoma, NOS	≈15	(3, 24, 60)
Burkitt lymphoma	≈60	(3, 24, 61)
Extranodal NK-/T-cell lymphoma	>90	(3, 24, 62)

EBV, Epstein–Barr virus; NOS, not otherwise specified.

The main route of EBV infection in lymphoid cells is through the CD21 receptor; however, this molecule is not expressed by -T/-NK lymphocytes. Therefore, it is believed that in -T/-NK lymphoproliferative disorders, EBV infects a lymphoid progenitor that expresses CD21, which subsequently differentiates into mature -T/-NK cells (66). Furthermore, intragenic EBV deletions are recurrently observed in chronic active EBV infection and in ENKTCL, which may represent important events for tumorigenesis (67). Additionally, certain EBV strains with a particular predisposition to infection and expansion of -T/-NK lymphocytes are more prevalent in Asia and South America, a fact that helps to explain the higher frequency of this lymphoma subtype in these geographic areas (68).

After infecting -T/-NK cells, viral oncoproteins, such as LMP-1, stimulate the constitutive activation of intracellular signaling pathways AKT, JAK/STAT (STAT3, JAK3, and STAT5B), MAPK, and nuclear factor kappa B (NF-κB), inhibiting apoptosis, promoting cell proliferation, and modulating the immune response, consequently regulating the interactions between the neoplastic compartment and the non-tumor immune microenvironment (69). Additionally, the LMP-1 oncoprotein promotes great genomic instability, inducing mutations and copy number alterations in several oncogenes, such as those located on the 6q21-q25 regions and in tumor suppressors, such as *TP53*, resulting in the development and progression of ENKTCL. Mutations in *DDX3X* RNA helicase gene and in *BCOR* gene, which encodes a co-repressor of the BCL-6 transcription factor, are also recurrently observed in ENKTCL, playing a fundamental role in its oncogenesis (38, 70, 71).

ENKTCL pathogenesis is strongly dependent on EBV oncoproteins, with almost all cases containing EBV genomes and encoded small RNA in neoplastic cells. Certainly, these findings provide potential targets for precision treatment (58–62). In ENKTCL lymphomagenesis, oncogenic events related to EBV are probably one of the earliest occurrences that trigger signal transduction activation, upregulation of antiapoptotic proteins like BCL2A1, and activation of various intracellular pathways. NF-κB, a transcription factor responsible for mediating the proliferation and survival of B and T cells hindering cell apoptosis and consequently promoting tumor evolution, is usually upregulated in ENKTCL (72). Alterations of the JAK/STAT pathway, especially *JAK3*, involving its mutations and aberrant phosphorylation are highly prevalent and relate to tumor cell survival (4, 73). PD-1 is an immune inhibitory receptor belonging to the CD28 family expressed by activated T and B cells, which plays an important role in tumor immune escape, with almost all EBV LDs being associated with high levels of PD-L1 expression (74). Activation of the STAT3 pathway and overexpression of LMP-1 induce the upregulation of PD-L1 in ENKTCL, highlighting the appealing treatment results with immune checkpoint inhibitors (70).

**Figure 3:**
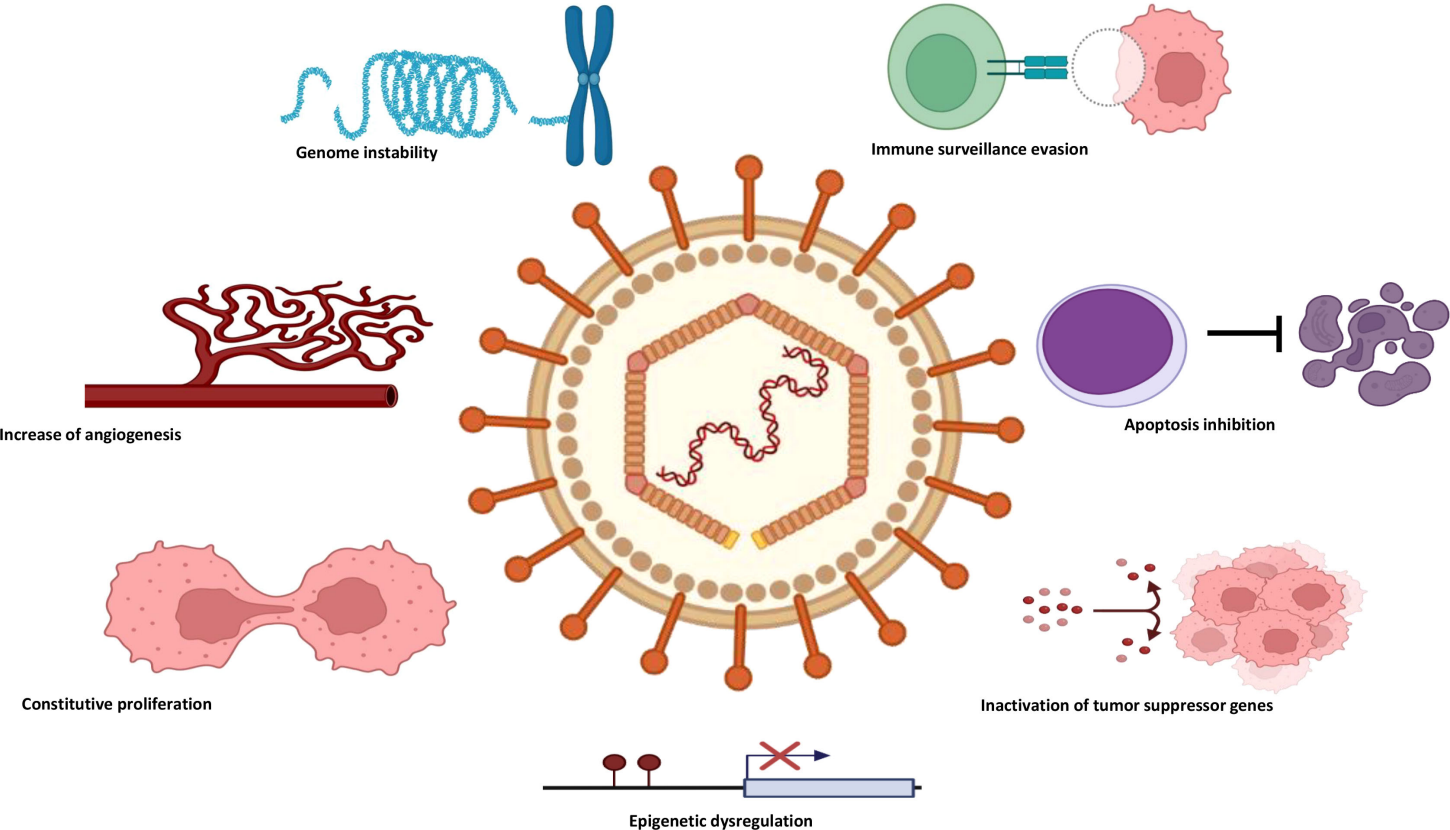
The main mechanisms of pathogenesis involved in ENKTCL. ENKTCL, extranodal NK-/T-cell lymphoma.

Both genetic and epigenetic factors are crucial for the pathogenesis of ENKTCL, with the latter particularly important during EBV-associated tumorigenesis (75). As stated before, EBV presents a key role as an epigenetic driver in EBV-associated cancers. EBV-encoded oncoproteins, such as LMP-1, LMP-2, and EBNA-3, modulate the host-cell epigenetic machinery, reshaping the viral and host epigenomes throughout host epigenetic modifiers, including DNA methyl transferases, histone methyl transferases, polycomb group proteins, and histone deacetylases (76–78). In addition, EBV-encoded miRNAs have epigenetic and regulatory mechanisms and regulate host-cell biology and the microenvironment, contributing to immune evasion and migration of EBV-infected cells (79, 80). In a recent study conducted by Peng RJ et al. involving whole-genome sequencing (WGS) of 27 EBV-positive NKTCL tumor samples, the authors found 0.45% (0.03%–1.06%) of similarity with the viral genome when aligned to the human and EBV reference genomes. Among the 27 viral genomes identified in these tumor samples, approximately 1,152 SNVs and 44.8 indels (<50 bp) were revealed per sample, particularly in the BPLF-1 and BDLF-2/3 hotspot regions (81). Furthermore, different EBV strains (type A and B) and the genetic sequence of LMP-1 present distinct distributions worldwide (81). **Figure 3** illustrates the main mechanisms of pathogenesis involved in ENKTCL.

### 4.3 Molecular pathways implicated in ENKTCL development and progression

ENKTCL molecular pathogenesis is very complex, and its main alterations involve several signaling pathways related to distinct biological functions. Among them, we found deregulation in pathways implicated in cell proliferation and survival, resistance to cell death, immune evasion, DNA repair, angiogenesis, and epigenetic control (82).

Several signaling pathways related to cell proliferation are activated in ENKTCL, often presenting gain-of-function mutations involving key genes. The JAK/STAT pathway plays a central role in ENKTCL development. Mutations involving *JAK3*, *STAT3*, and *STAT5B* genes, as well as phosphorylation of the pseudokinase domain of the *JAK3*, found in up to 40% of cases, lead to constitutive activation of the JAK/STAT pathway and consequent pro-proliferative activity (83, 84). Additionally, *PTPRK* gene, located on chromosome 6q, physiologically inactivates the STAT3 protein. However, in ENKTCL, this gene is usually inactivated by deletion or hypermethylation of its promoter region, leading to consequent activation of the JAK/STAT pathway (85).

Increased expression of genes related to the NF-κB pathway has been demonstrated in ENKTCL (86). This pathway is involved in pro-proliferative activity in several lymphoid malignancies. Furthermore, the NF-κB pathway has recently been implicated in the genesis of hemophagocytosis, a complication recurrently observed in ENKTCL (53). *DDX3X* inactivating mutations, found in up to 50% of ENKTCL, are also associated with proliferative activity via transcriptional activation of the NF-κB pathway (51). Other pathways related to proliferation and cell cycle control, such as *C-MYC*, *RUNX3*, *NOTCH1*, and *Aurora kinase*, have recently been implicated in ENKTCL oncogenesis and seem to play an important role in the proliferation and survival of its neoplastic cells (82).

Evasion of mechanisms associated with programmed cell death strongly contributes to clonal cell viability in ENKTCL. Different mechanisms are associated with apoptotic escape in this malignancy, including survivin overexpression, deregulation, and/or mutations of *TP53* tumor suppressor gene, as well as reprogramming of cellular metabolism and mechanisms associated with autophagy (82, 87, 88). Evasion of the host immune system appears to be another mechanism used by ENKTCL tumor cells to survive and subsequently proliferate. Recent studies have demonstrated overexpression of PD-L1 by immunohistochemistry in ENKTCL tumor samples. Additionally, constitutive activation of the STAT3 pathway and overexpression of the viral oncoprotein LMP1 induce upregulation of PD-L1 in ENKTCL, contributing to the tumor escape from cytotoxic T-cell activity, which has been an important biological rationale to support the development of tests with immune checkpoint inhibitors in relapsed and refractory ENKTCL (70, 89, 90).

Dysregulation of the DNA damage response is also implicated in the genesis of ENKTCL, a malignancy associated with high genomic instability. Alterations involving the *ATM/ATR* axis (*ataxia telangiectasia mutated/related*), central regulators of the response to genomic damage, have been recurrently observed in a subgroup of patients with ENKTCL. *ATM/ATR* axis gene deletions, as well as mutations involving genes related to cell cycle checkpoint, are the main mechanisms implicated in the defective response to DNA damage observed in ENKTCL (91, 92).

ENKTCL is a highly vascularized tumor and is associated with a markedly angiocentric histopathological pattern. Consequently, pathways involved in neoangiogenesis seem to play a fundamental role in the survival of tumor cells and consequent progression of this neoplasm. In this sense, recent studies indicate increased expression of genes and proteins related to angiogenesis in ENKTCL samples. Among these, the *vascular endothelial growth factor-A* (*VEGF-A*), its receptor *VEGFR2*, the *hepatocyte growth factor* (*HGF*), and its receptor *MET* stand out (38, 93).

Pathways related to epigenetic modulation are also deregulated in ENKTCL. Mutations involving several epigenetic regulators have been recurrently found in ENKTCL cases, with emphasis on mutations involving the *BCOR*, *MLL2*, *ASXL3*, *ARID1A*, and *EP300* genes. These discoveries have served as a biological rationale for incorporating epigenetic-modulating drugs, such as histone deacetylase inhibitors (HDACi), into the list of new agents to be tested for ENKTCL (51, 94, 95).

Even though a better understanding of the pathogenesis of ENKTCL is evolving, it not always translates into clinical practice changes. One example is one of the earliest clinical trials using bortezomib, a proteasome inhibitor that prevents NF-κB activation. Promising results were initially demonstrated in association with the CHOP (cyclophosphamide, doxorubicin, vincristine, and prednisone) regimen, but dismal outcomes were obtained in the more modern chemotherapy association scheme era (96, 97). It is of most importance to combine molecular advances obtained in the ENKTCL recent studies with the appropriate selection of patients together with the best therapeutic combinations in clinical trials. **Table 3** summarizes the main mechanisms associated with ENKTCL pathogenesis.

**Table 3:** Main mechanisms associated with ENKTCL pathogenesis.

Pathogenic mechanism	Details
Chromosomal aberrations	Gains at chromosomes 1p, 6p, 11p, 12q, 17q, 20q, and Xp Losses at chromosomes 6q, 11q, 13q, and 17p Del(6q-) are associated with losses of tumor suppressor genes *FOXO3*, *HACE1*, *PRDM1*, *ATG5*, and *AIM1* Del(17p-) is associated with loss of the tumor suppressor gene *TP53*
EBV-mediated oncogenesis	Viral oncoproteins LMP-1 and EBNAs Intragenic EBV deletions More pathogenic EBV strains—prevalent in Latin America and Asia Activation of proliferative signaling pathways: JAK/STAT, AKT, MAPK, and NF-κB; apoptosis inhibition and immune modulation via PD-1/PD-L1 axis
Deregulated molecular signaling pathways and mutational landscape	Pro-proliferative effect and cell-cycle regulation: JAK/STAT, NF-κB, *C-MYC*, *RUNX3*, NOTCH1, and Aurora kinase pathways Apoptosis inhibition: surviving overexpression, deregulation of *TP53* pathway, and reprogramming of cellular metabolism and autophagy Immune evasion: PD-1/PD-L1 axis Dysregulation of DNA repair: ATM/ATR axis Pro-angiogenic activity: *VEGFA*, *VEGFR2*, *HGF*, and *MET* Epigenetic dysregulation: *BCOR*, *MLL2*, *ASXL3*, *ARID1A*, and *EP300* Mutational landscape: *STAT3*, *JAK3*, *STAT5B*, *SOCS1*, and *PTPRK* (JAK/STAT pathway ~40%) *MAP3K5*, *BRAF*, and *EPH1A* (RAS/MAPK pathway ~15%) *ECSIT*, *IKBkB*, and *BIRC2* (NF-κB pathway ~15%) *DDX3X* (RNA helicases, ~40%–50%), *TP53* (~10%) *BCOR*, *EP300*, *TET2*, and *ARID1A* (epigenetic machinery, ~5%–10%)

ENKTCL, extranodal NK-/T-cell lymphoma; EBV, Epstein–Barr virus.

## 5 Molecular features, classification, and clinical applicability

As previously mentioned, several tumor suppressor genes located on chromosome 6q21-q25 are inactivated by the deletion of this locus, an event recurrently found in ENKTCL (32). Among these, the main ones are *FOXO3* and *PRDM1*. *FOXO3* is a *forkhead* family transcriptional factor implicated in the induction of apoptosis and cell cycle arrest in NK cells, while *PDRM1* regulates NK-cell activation and maturation (98, 99). Additionally, deletion or mutation of the tumor suppressor *TP53* has been observed in more than 30% of ENKTCL cases (100).

Genes implicated in the regulation of several signaling pathways are recurrently mutated in ENKTCL. Studies using next-generation sequencing (NGS) have shown that more than 40% of ENKTCL cases present mutations involving genes with a fundamental role in epigenetic regulation and the JAK/STAT pathway (101). Among the epigenetic modifiers, the most commonly mutated genes are *MLL2*, *MLL3*, *BCOR*, *TET2*, *EP300*, and *ARID1A*. *MLL2* and *MLL3* belong to the *KMT2* family and participate in nuclear chromatin remodeling (48). *BCOR* encodes the co-repressor for the transcription factor BCL-6 and is involved in histone modification. BCOR gene silencing promotes cell proliferation and activation of the AKT pathway (49). *TET2*, *EP300*, and *ARID1A* mutations are less frequent, occurring in approximately 7%–10% of ENKTCL cases. JAK/STAT pathway gene mutations particularly affect *STAT3*, *JAK3*, *STAT5B*, *SOCS1*, and *PTPRK* genes. This pathway is crucial for the development and maturation of NK cells. Such mutations usually lead to the constitutive activation of the JAK/STAT pathway, promoting the growth, survival, and migration of tumor cells (47).

Mutations involving RAS-MAPK pathway genes are found in up to 15% of ENKTCL, commonly affecting *MAP3K5*, *BRAF*, and *EPH1A* genes, as well as NF-κB pathway activating mutations involving *ECSIT*, *IKBKB*, and *BIRC3* genes. Inactivation of RNA helicases, a negative regulator of NK-cell proliferation, usually occurs by mutations of *DDX3X* gene and less frequently by mutations involving *SHX58*, *DDX18*, and *DDX21* (51). Some mutations were associated with prognoses in ENKTCL, such as mutations in *DDX3X*, *TP53*, and *KMT2D*, which were correlated with decreased survival (50).

A recent experimental study based on phenotypic and molecular analyses of ENKTCL demonstrated tumor cell arrest at the early stages of NK maturation, suggesting that its COO is not a terminally differentiated NK cell. Additionally, ENKTCL neoplastic cells demonstrated genome-wide DNA hypermethylation, particularly in polycomb-marked regions. Such alterations were associated with extensive gene silencing, loss of transcriptional factor binding, and overexpression of *EZH2*, particularly in epigenetically more immature tumors. Based on the demonstration of this globally hypermethylated phenotype in ENKTCL, the authors investigated the potential therapeutic applications of the hypomethylating agent 5-azacytidine in a xenograft model inoculated with ENKTCL cells. The treatment led to the re-expression of NK-cell developmental genes, phenotypic NK-cell differentiation, and prolonged survival, opening precedents for the potential therapeutic application of epigenetic modifiers in this lymphoma subtype (102).

In a pioneering way, recently, Xiong et al. proposed to classify ENKTCL in three molecular subtypes based on multi-omic data, as summarized in **Table 4**. In this study, genomic and transcriptome analyses were performed in 128 biopsies of ENKTCL (27). The authors described the following molecular subgroup of ENKTCL: tumor suppressor–immune modulator (TSIM), MGA-BRDT (MB), and HDAC9-EP300-ARIDIA (HEA). The TSIM is characterized by deletion of chromosome 6q21, containing tumor suppressor genes, 9p24.1/*PDL1/2* overexpression, *JAK-2* amplification, 17q21.2/*STAT* amplification, JAK/STAT pathway mutation, *p53* mutation, increased expression of NK cell-associated immunity, defected immune responses associated with inappropriate antigen processing and presentation, and genomic instability. TSIM also presented higher NK gene expression, while HEA presented higher T-cell gene expression. The TSIM presented an EBV latency type II and a higher level of lytic gene *BALF3*. The MB molecular subgroup presented LOH in 1p22.1/*BRDT* and *MGA* mutations related to the upregulation of *MYC*, *MAPK*, *NOTCH*, and WNT signaling pathways, with EBV latency type I. The HEA subtype presents EBV latency patterns type II, with high levels of lytic gene *BNRF1* and is characterized by mutation of epigenetic modifiers with activation of NF-κB pathway and TCR signaling pathways. In general, the MB subtype has a worse outcome when compared to TSIM and HEA subtypes. The TSIM molecular subgroup represents the prototype of ENKTCL, typically of the NK lineage. However, the MB may correspond to ENKTCL of the T-cell lineage and represents the worst prognostic group with increased expression of *MYC*, resulting from a silencing mutation of *MGA* (27, 101). Interestingly, this molecular classification has important prognostic and therapeutic implications. While the MB subtype is associated with poor survival, the HEA group has an estimated 3-year overall survival (OS) above 90%. Likewise, individuals from the HEA group have a biological rationale for the therapeutic use of HDACi, while those with the TSIM subtype may be managed with JAK inhibitors (ruxolitinib) and/or immune checkpoint inhibitors, such as nivolumab/pembrolizumab (27, 101).

**Table 4:** Molecular subtypes of ENKTCL.

	TSIM subtype	MB subtype	HEA subtype
Main genomic alterations	Mutations in JAK/STAT and p53; amp9p24.1/JAK2 locus; amp17q21.2/STAT3/5B/5A locus. amp9P24.1/PD-L1/2 locus, del6q21(	MGM mutation and 1p22.1/BRDT LOH)	Mutations in HDAC9, EP300 and ARID1A genes
NK gene expression	+++	++	++
T-cell gene expression	++	++	+++
EBV gene expression pattern/latency	High BALF3/type II latency	Low LMP1/type I latency	High BNFR1/type II latency
Myc overexpression	–	+++	–
Signaling Pathway activated	JAK/STAT (JAK2/3, STAT3, and STAT5B)	NOTCH, MAP WNT (NOTCH3, MAP3K6, WNT2/11)	NF-κB
3-year overall survival	79.1%	38.5%	91.7%

Adapted from Xiong et al. (2020) (27).

TSIM, tumor suppressor–immune modulator; MB, MGA-BRDT; HEA: HDAC9-EP300-ARIDIA; ENKTCL, extranodal NK-/T-cell lymphoma; EBV, Epstein–Barr virus.

-, abscense of expression; +, low expression; ++, moderate expression; +++, high expression.

## 6 Staging and prognostic factors

The Lugano classification, derived from the Ann Arbor staging system, although routinely used for ENKTCL, lacks utility in prognostication, as these lymphomas are extranodal in origin, and this classification does not consider the adverse prognostic impact of extranasal anatomical sites (103). Different staging systems that consider local tumor invasion, disease spread pattern of local structures, lymph node, or distant sites involvement have been proposed with better accuracy, but they are not as simple as the conventionally used Ann Arbor system, which seems hard to be replaced in clinical practice (104, 105). Early-stage disease is considered nasal stage IE or contiguous stage IIE (cervical node involvement). All cases that are extranasal in origin are considered advanced-stage, with the rare exception of stage IE based on cutaneous involvement, which should be classified as a localized disease after a thorough staging for treatment purposes (2, 106). Regarding imaging modalities for staging, the high accuracy of ^18^F-fluorodeoxyglucose–positron emission tomography/computed tomography (^18^F-FDG-PET/CT) in ENKTCL has been demonstrated and should be included in the initial assessment (107, 108). In one of the largest cohorts in ENKTCL, ^18^F-FDG-PET/CT detected 58 nodal and 69 extranodal lesions versus 44 and 61 detected by conventional methods, respectively (p < 0.001). Of note, in this study, 21.2% and 44.2% of patients had disease stage and treatment planning changed, respectively, with 97.7% sensitivity for PET/CT and 80.7% for conventional methods (109). Also, cutaneous and bone marrow infiltration by ^18^F-FDG-PET/CT has higher sensitivity when compared to conventional methods such as bone marrow biopsy, making ^18^F-FDG-PET/CT the modality of choice for staging (110, 111).

Additionally, prognostic information can be obtained by maximum standardized uptake value (SUV) analysis on diagnosis with worse outcomes seen in patients with higher SUV uptake (SUVmax > 15). Other adverse factors, such as bulky disease and local invasion, are independent prognostic factors for decreased progression-free survival (PFS) and OS (112). Other recent proposed refinements of ^18^F-FDG-PET/CT are whole-body metabolic tumor volume (MTV) and whole-body level of total lesion glycolysis (LTLG). Therefore, combining tumor size with metabolic activity seems a promising prognostic tool; however, it was validated only in clinical trials and is not incorporated into clinical practice, even in much more common lymphoma subtypes (113). Noteworthy, patients from Asia are the most represented in ENKTCL studies, usually with a very short follow-up, making standardization and reproducibility a challenge, especially in middle-income countries, another geographically relevant area in this topic (114). International Prognostic Index (IPI), the most used predictive model in NHL, fails in accuracy for ENKTCL, as most patients have localized disease and good performance status and are classified as low risk by IPI; therefore, the Korean Prognostic Index (KPI) was proposed. Although KPI performed better than IPI, it was validated in most patients on anthracycline-containing regimens, and as newer treatments emerge, new prognostic factors are of paramount importance (115, 116).

Since some studies correlate pre-treatment EBV plasmatic viral load with response to treatment and overall survival, EBV serum viral load was incorporated into some prognostic scores (117). One recently proposed and useful prognostic index is the Prognostic Index for Natural Killer Lymphoma (PINK) and its variant, the Prognostic Index for Natural Killer Lymphoma plus EBV (PINK-E) scores, built from a large cohort (n = 527) of non-anthracycline-treated patients using clinical parameters (PINK) and clinical parameters plus EBV DNA data from the same cohort (n = 328 for PINK-E) (118). In this study, the authors found that age > 60 years, non-nasal type ENKTCL, distant node involvement, and advanced-stage disease adversely affect the prognosis. The patients were stratified into low risk (no risk factors), intermediate risk (one risk factor), or high risk (two or more risk factors) with 3 years OS of 81%, 62%, and 25%, respectively. The 328 patients with data for EBV DNA were stratified into three categories with different rates of overall survival. Although EBV measurements are not universally available and reference values are not standardized, PINK-E is the most reliable prognostic tool and should be adopted in clinical practice. Another promising application is the use of circulating EBV DNA as a biomarker for minimal residual disease and early relapse detection (119). In this sense, plasmatic EBV DNA measured in the interim of treatment, usually after at least two cycles of chemotherapy, has shown an important association with clinical outcomes, as well as interim imaging evaluation with ^18^F-FDG-PET/CT. Different studies have demonstrated that ENKTCL patients with undetectable viral load and complete metabolic response (Deauville score <3) in the interim of primary therapy have markedly increased survival in comparison to patients who do not reach such goals. These same parameters are also capable of predicting an adverse prognosis if it remains positive at the end of treatment (120–122).

Aiming to overcome the limitations imposed by ENKTCL prognostic scores based on clinical and laboratory parameters, Tian XP et al. developed a molecular prognostic score based on the presence of seven single-nucleotide polymorphisms (7-SNP score). The selected seven SNPs were related to *WDR27*, *UMAD1*, *TENM2*, *LINC02463*, *KDM4C*, *FGD4*, and *FAM71A* genes. Data from 722 patients with ENKTCL from different regions of the world were analyzed and allocated into a training cohort, an internal validation cohort, and two external validation cohorts. Patients with low-risk and high-risk scores by this classifier exhibited significantly different OS and PFS (p < 0.001) (123). Although this score has shown high accuracy in the ENKTCL prognostic stratification, it incorporates a costly, poorly available, and complex methodology. Therefore, it has not proved to be feasible to replace PINK and PINK-E in clinical practice (124).

Recently, a Chinese multi-institutional study proposed the creation of a nomogram-revised risk index (NRI) based on the selection of risk variables obtained in a multivariate analysis of a previous cohort composed of 1,383 ENKTCL patients (125). Subsequently, the results were validated in a cohort of 1,582 cases undergoing treatment not based on anthracyclines. The variables included in the NRI were age ≥ 60 years, Eastern Cooperative Oncology Group (ECOG) score ≥2, high lactate dehydrogenase (LDH) levels, local primary tumor invasion (PTI) or stage II (1 point for each), and advanced-stage III/IV (2 points). Patients were stratified into five groups (low, low-intermediate, high-intermediate, high, and very high) with markedly different estimated 3- and 5-year OS. The NRI showed better performance for predicting OS than the IPI, KPI, and PINK prognostic scores. Such data indicate NRI as a promising and effective tool for predicting prognosis in ENKTCL, as well as for the appropriate selection of patients for individualized therapeutic strategies (126).

## 7 Treatment

As in other aggressive lymphomas, ENKTCL is a type of potentially curable tumor, and all fit patients should be treated with curative intention. The most important factors regarding the choice of treatment are the stage of disease and performance status; although with novel therapies emerging, these current concepts of treatment may change in the near future. Because of the rarity of this NHL subtype, no standard treatment based on well-designed randomized trials is available. One fundamental concept is that, apart from other B-cell aggressive lymphomas, ENKTCL tumor cells have a high expression of multidrug resistance (MDR) gene *ABCB1* and its product, P-glycoprotein (Pgp), which can partly explain the poor outcomes when conventional schemes based on anthracyclines are used (127).

### 7.1 Limited-stage newly diagnosed disease

Historically, given the very poor outcomes with anthracycline-based chemotherapy alone (CHOP regimen) and given the radiosensitivity of ENKTCL, extended field radiotherapy (EF-RT) became a cornerstone for early-stage disease, and its omission has, since then, showed a negative impact in several studies (128, 129). Nevertheless, radiotherapy alone is not sufficient due to high rates of relapsed disease outside the radiation field (130, 131), and for limited stages, IE to IIE with nodal involvement disease combined modality therapy (radiation therapy with chemotherapy) is the standard of care. In the rare exception of stage IE primary cutaneous disease, RT alone can be considered due to anecdotal cases with more indolent clinical behavior (2, 106).

Although previous studies indicated that RT doses <50 Gy were associated with inferior response rates, more recent trials have shown that lower doses as 40–44 Gy in association with combined modalities may offer the same outcomes with good local control (132, 133). However, for clinically unfit patients unable to receive combined chemoradiotherapy (CRT), an adequate radiation dose would be >50 Gy. In combined modalities, RT doses should be given according to those established in the protocol of choice. While there is uncertainty regarding how to sequence these two modalities, a meta-analysis showed survival benefits for patients treated with RT upfront (5). Concurrent chemoradiotherapy, sequential chemoradiotherapy, and “sandwiched” chemoradiotherapy have been evaluated in prospective studies, and the choice may depend on the prompt access to RT. One commonly used RT first regimen is the RT-2/3 DeVIC (dexamethasone, etoposide, ifosfamide, and carboplatin) therapy protocol, published by the Japan Clinical Oncology Group. In this phase I/II trial, concurrent RT (50 Gy) plus carboplatin for 6–8 weeks was followed by three cycles of dexamethasone, etoposide, and ifosfamide. An updated analysis of this study revealed a PFS of 67% and an OS rate of 73% at 5 years. Although grade 3–4 hematological toxicities were observed in all patients, neutropenia was manageable, and the most common late toxicities were radiation-related, with no second malignancies reported. Of note, only patients with a performance status of 0 to 2 were included in this trial (134, 135).

The Korean group evaluated RT-cisplatin followed by VIPD (etoposide, ifosfamide, cisplatin, and dexamethasone) protocol. In this study, cisplatin as a single agent was given weekly during radiation (median 40 Gy dose), followed by three cycles of VIPD (etoposide, ifosfamide, cisplatin, and dexamethasone) after 3–5 weeks of RT. In this study, the PFS and OS rates reported were 85.1% and 86.2%, respectively. With grade 3–4 hematologic toxicities highly observed during VIPD, there were only two infection-related deaths (2/30 patients) (136). A recent observational study from South America reported inferior outcomes and toxicities with the same protocol in the real-world setting, with 57.1% (12/21) deaths during the induction phase and a 2-year OS of 53.2%. This probably reflects selection bias, as patients in clinical practice have poorer PS and may have comorbidities and infections, and these differences must be considered in clinical practice (128). Sequential and “sandwiched” chemoradiotherapy involve chemotherapy followed by interim RT. Different protocols, most using asparaginase-containing regimens, are under use with outstanding overall response rates (ORRs) >90% (137, 138). As no randomized trials comparing concurrent or sequential CRT exist, all proposals are adequate for limited-stage disease, and decisions must encompass individual and logistic issues, such as infection control, radiation availability, as previously mentioned, and even distance from the tertiary hospitals, especially in low- or middle-income countries.

### 7.2 Advanced-stage and relapsed/refractory disease

Approximately 25%–30% of ENKTCL patients are diagnosed with advanced-stage disease, and for those patients, although they have poor prognoses, more recent data suggest better responses for patients treated in the modern era when compared to anthracycline-based chemotherapy. In a recent large real-world retrospective analysis of 2,560 ENKTCL patients from China, 334 (13%) patients were advanced-stage, and treatment was dichotomized into non-anthracycline-based therapy (non-ANT) *vs.* ANT-based regimens, with superior PFS and OS favoring non-ANT regimens (139). Notably, an intensive treatment containing asparaginase-based chemotherapy enhances responses and is currently the standard of care as shown in a meta-analysis (140). Chemotherapy schemes vary by institution, but one of the most used is the SMILE protocol, which consists of methylprednisolone, methotrexate, ifosfamide, l-asparaginase, and etoposide, given every 28 days, for two cycles or more. In a prospective study, of 38 patients enrolled, 74% were able to complete at least two cycles. With a median follow-up of 2 years, the ORR and complete response (CR) rate were 79% and 45%, with 55% and 53% OS and PFS, respectively (141). Although chemotherapy alone is the current practice, the role of radiation in advanced disease is not well established but may be beneficial for patients to achieve a complete response after chemotherapy. A retrospective analysis of advanced-stage disease showed a 2-year OS rate of 81.5% for post-chemotherapeutic RT patients *vs.* 40.2% for those not irradiated (142). Conversely, in another retrospective analysis of 102 advanced-stage disease patients, 23 received adjuvant radiation with no benefit in OS (p = 0.91), and of note, in this study, the best response rate was achieved with asparaginase-based therapy (SMILE), when compared to CHOP or DeVIC-like regimens (143).

Although the SMILE regimen is widely used in the treatment of advanced-stage ENKTCL, its toxicity is not negligible, particularly with regard to cytopenias and the occurrence of infectious complications. In order to minimize such adverse effects, Chinese researchers proposed the DDGP regimen (dexamethasone, cisplatin, gemcitabine, and peg-asparaginase) as an alternative to the SMILE protocol for managing ENKTCL in stages III/IV, relapsed/refractory disease, or extranasal disease. Consequently, in 2016, Xin Li et al. reported the results of a phase 3, randomized, multicenter study involving 42 Chinese patients with advanced-stage ENKTCL and ECOG ≤ 2. Patients underwent primary therapy with the DDGP (N = 21) or SMILE (N = 21) protocols. ORR and CR were increased in the DDGP arm (95% *vs.* 67% for ORR, p = 0.018; 71% *vs.* 29% for CR, p = 0.005). Similarly, 1-year PFS and 2-year OS were better in the DDGP group than in SMILE (86% *vs.* 38% for 1-year PFS, p = 0.006; 74% *vs.* 45% for 2-year OS, p = 0.027). At the same time, the group treated with SMILE developed a higher rate of adverse events, including leukopenia and allergic reactions (144). Later studies confirmed the results found in this trial, pointing to the DDGP regimen as a therapeutic strategy associated with higher response rates, increased survival, and better tolerability than SMILE chemotherapy for the treatment of patients with advanced-stage ENKTCL or with relapsed/refractory (R/R) disease (145, 146).

Since upfront autologous hematopoietic stem cell transplantation (AHSCT) results in similar response rates when compared to CCRT treatments for limited-stage disease, the current guideline from the *American Society for Blood and Marrow Transplantation* recommends against upfront AHSCT in newly diagnosed localized ENKTCL patients who achieve CR with modern therapy (147, 148). Furthermore, patients with advanced-stage disease do not seem to benefit from autologous hematopoietic cell transplantation (HCT) when treated with asparaginase-based regimens. A retrospective study demonstrated a 3-year PFS and OS of 40.1% and 52.3%, respectively, for the advanced-stage disease cohort, and patients in partial response (PR) did not benefit from the therapy, with a 3-year PFS of 13.3%. In multivariate analysis, pre-transplant PR and anthracycline-based primary chemotherapy were independent prognostic factors for reduced PFS. For OS, anthracycline-based primary chemotherapy was the only independent factor for increased risk of death (149). For patients who achieve CR and have adverse clinical variables (high prognostic score index), a retrospective analysis demonstrated notably improved survival, making AHSCT a post-induction consolidation choice for selected patients. However, more studies are needed (148).

Relapsed or refractory patients are often included in the same studies designed for advanced-stage disease, and clinical trials are the preferred treatment option after treatment with asparaginase-based chemotherapy. SMILE, AspaMetDex (asparaginase, methotrexate, and dexamethasone), P-GEMOX (peg-asparaginase, gemcitabine, and oxaliplatin), and GDP (gemcitabine, dexamethasone, and cisplatin) trials all included relapsed or refractory patients, but prognosis in this population is very poor (141, 150–152). Of note, in SMILE clinical trial, patients in the first relapse had a CR of 46%, but no refractory patient achieved CR (141). Allogenic HSCT results were reported by the *Center for International Blood and Marrow Transplant Research* (CIBMTR) study group with discouraging results. In this analysis, the 2-year PFS and OS were 20% and 24%, respectively, with a PFS of only 20% even in CR patients prior to transplant (153). A large recent analysis from the *National Spanish Group* reported the outcomes of allogenic HSCT in advanced mature T- and NK/T-cell neoplasms (6.5% ENKTCL) and showed 1-year non-relapse mortality of 21.9%, mainly due to graft-versus-host disease (GVHD) and bacterial infections (154). In addition, the current guideline from the *American Society for Blood and Marrow Transplantation* has a weak recommendation for allogeneic HSCT in this setting (147). Therefore, this group of patients represents an unmet medical need since most will not even be eligible for HSCT for inadequate functional status, organ dysfunction, comorbidities, and uncontrolled disease status, making them candidates for new agents in development.

### 7.3 Novel therapies

Initial impressive results involving immune therapies, especially immune checkpoint inhibitors as single-agent therapy, have been published since PD-L1 is expressed in ENKTCL (155). In one study, seven patients previously treated with asparaginase-based regimens (two with allogenic HSCT) received a median of seven cycles of the anti-PD-1 antibody pembrolizumab with an ORR of 100%, and five of them achieved CR (156). One year later, the same group reported the efficacy of another PD-1 inhibitor, nivolumab, in low doses (157). Tislelizumab in combination with chemotherapy has also proven feasible, and clinical trials with this immune checkpoint inhibitor are ongoing for both early- and advanced-stage diseases in combination with RT or chemotherapy (158, 159). Therefore, blockade of the PD-1/PD-L1 immune axis has been shown to be a safe and effective option in the management of R/R ENKTCL. However, there seems to be no direct correlation between PD-L1 antigen expression in tumor cells and therapeutic response to immune checkpoint inhibitors (160). Currently, no predictive factors of response to these drugs have been identified in ENKTCL.

In addition to initial studies demonstrating the efficacy of pembrolizumab and nivolumab in patients with ENKTCL R/R to asparaginase-based chemotherapy, new trials have evidenced the efficacy of other anti-PD-1 and anti-PD-L1 antibodies, such as sintilimab and avelumab, respectively (156, 157). In the ORIENT-4 trial, 28 patients with R/R ENKTCL received sintilimab at a dose of 200 mg I.V. every 3 weeks for 24 months. With a median follow-up of 30.4 months, the median OS was not reached, and the estimated 2-year OS was 78.6%. Serious adverse events occurred in only 25% of cases, and no patient died from toxicity. Thus, sintilimab proved to be a safe and effective therapeutic strategy for the management of R/R ENKTCL (161). Similarly, a recent phase 2 study evaluated the efficacy and safety of the anti-PD-L1 antibody avelumab in 21 cases of R/R ENKTCL. In this study, the responses were lower, with an ORR of 38% and a CR of 24%. No grade 4 adverse effects occurred, but there was a correlation between response and tissue expression of the PD-L1, with all patients who achieved CR presenting high PD-L1 expression (162). Although it had moderate activity as a single agent, avelumab seems to be an interesting option for trials testing it in association with other drugs, with particular potential benefit in cases of R/R ENKTCL with a high density of the PD-L1 antigen.

A proportion of ENKTCL express the transmembrane glycoprotein receptor CD30 (Ki-1) and CD38, and the clinical activity of antibodies directed against both antigens has been evaluated as possible target therapies alone or in combination. A phase 2 study with daratumumab monotherapy, a monoclonal antibody targeting CD38, demonstrated a 25% ORR in relapsed/refractory ENKTCL patients. Although feasible, no patient achieved CR, and patients presented a short duration of response (163).

Although there is a biological rationale for the use of JAK/STAT inhibitors in ENKTCL, few studies have assessed the real impact of these agents on this neoplasm. *In vitro* studies have shown that the use of the pan-JAK inhibitor CP690550 and the JAK2 inhibitor AG490 resulted in decreased phosphorylation of STAT3 and STAT5 and subsequent stimulation of apoptosis in ENKTCL cell lines (47, 164). Tofacitinib, a pan-JAK inhibitor with higher JAK-3 selectivity, has demonstrated activity, inducing cell cycle arrest and growth inhibition in both positive and negative EBV NK cell lines and may be an attractive therapy (165). Currently, an ongoing phase 2 study (NCT03598959) is evaluating the safety and efficacy of the combination composed of the pan-JAK inhibitor tofacitinib with chidamide in R/R ENKTCL.

HDACi also have a potential therapeutic effect on ENKTCL, particularly on the HEA molecular subgroup, which is enriched in mutations involving epigenetic regulators. Supported by this biological principle, monotherapy with chidamide, a selective HDAC 1, 2, and 3 inhibitor, has been tested in a phase 2 trial involving 15 patients with R/R ENKTCL. In monotherapy, its activity was modest, with CR achieved in only 33% of cases and with a short median duration of response (166). However, this agent is currently being tested in combination with other drugs.

The oncogenic EBV protein overexpression may be a target for adoptive immunotherapy with antigen-specific cytotoxic cells, and this strategy was explored as a post-remission therapy with promising results (167). In addition, the association of nanatinostat with valganciclovir showed promising results in a phase 1/2 trial, and ENKTCL patients refractory to their last therapy presented an ORR of 60%, including 27% of CR (168). Although chimeric antigen T-cell receptor (CAR-T) therapy has been proven to be an interesting form of immunotherapy in several B-cell lymphoid malignancies, such as R/R diffuse large B-cell lymphoma, follicular lymphoma, B-cell acute lymphoblastic leukemia, and multiple myeloma, its use has been extremely limited in the setting of -T/-NK lymphoid disorders. While encouraging results were seen with some novel agents, there are still very little data about CAR-T in PTCL; limited experience and lack of controls preclude critical analyses. One of the main challenges in the use of CAR-T therapy in -T/-NK cell malignancies is due to the fact that neoplastic cells share a series of common antigens with normal T-lymphocytes, which can lead to fratricide and serious T-cell lymphoid aplasia in the receptor. To mitigate this effect, a selection of appropriate antigenic targets is essential. Currently, specific antigens have been selected for the construction of chimeric products, among which CD30, CD37, TRBC1, CCR4, and CCR9 stand out. The use of nanobody-derived or naturally selected CAR-T is an attractive strategy to overcome fratricide. Another problem intrinsic to the use of this therapeutic modality in T-cell malignancies refers to the potential contamination of the product collected for the construction of CAR-T with clonal T-cells; however, the use of allogeneic CAR-T products or CAR-NK-cells is a possible strategy with the ability to mitigate this contamination (169). Currently, data about the use of CAR-T therapy in ENKTCL are very scarce, although it may constitute an interesting therapeutic option for R/R disease in the near future. A trial with anti-CD30 CAR-T is underway for the treatment of R/R CD30-positive PTCL (NCT03049499) and its results are being eagerly awaited by the scientific community. **Table 5** summarizes the main therapeutic options for ENKTCL patients in different clinical settings.

**Table 5:** Main strategies adopted for the up-front management of ENKTCL, R/R disease, and novel therapies.

ENKTCL clinical presentation	Main therapeutic strategies
1. Early stage (IE-IIE)	1.1. Very elderly (≥80 years old), unfit cases, or primary cutaneous presentation • Isolated EF-RT with 50–60 Gy 1.2. Fit patients • Chemoradiotherapy (CRT)—concurrent, sequential, or *sandwiched* modalities - 2 × P-GEMOX/EF-RT/2 × P-GEMOX - RT plus weekly cisplatin followed by 3 × VIPD (Korean protocol) - RT followed by 2/3 DeVIC
2. Advanced-stage (III-IV) or extranasal disease (except cutaneous localized disease)	- 2/3 × SMILE - 6 × DDGP - 4/6 × AspMetDex - 4 × P-GEMOX - 4/6 × GDP * Consider up-front consolidation with ASCT for cases presenting high-risk PINK or PINK-E scores
3. Relapsed/refractory (R/R) disease	- Anti-MDR-based chemotherapy (SMILE, DDGP, and P-GEMOX) followed by ASCT consolidation or allo-SCT (in cases previously submitted to ASCT)
4. Novel therapies	- Clinical trials - Anti-PD-1/PD-L1: nivolumab, pembrolizumab, tislelizumab, sintilimab, avelumab - Anti-CD30: brentuximab-vedotin - Anti-CD38: daratumumab - JAK/STAT inhibitors: ruxolitinib, tofacitinib - HDACi: chidamide - Antiviral agents: valganciclovir plus nanatinostat - CAR-T therapies

ENKTCL, extranodal NK-/T-cell lymphoma; EF-RT, extended-field radiotherapy; CRT, chemoradiotherapy; RT, radiotherapy, P-GEMOX, peg-asparaginase, gemcitabine, and oxaliplatin; VIPD, etoposide, ifosfamide, cisplatin, and dexamethasone; DeVIC, dexamethasone, etoposide, ifosfamide, and carboplatin; SMILE, methylprednisolone, methotrexate, ifosfamide, l-asparaginase, and etoposide; DDGP, dexamethasone, cisplatin, gemcitabine, and peg-asparaginase; AspMetDex, l-asparaginase, methotrexate, and dexamethasone; GDP, gemcitabine, dexamethasone, and cisplatin; ASCT, autologous stem cell transplantation; allo-SCT, allogeneic stem cell transplantation; HDACi, histone deacetylase inhibitors; CAR-T, chimeric antigen T-cell receptor.

## 8 Conclusion and future directions

Successfully, the last decade brought us a better understanding of the importance of combined modality therapy and the relevance of asparaginase-containing protocols for ENKTCL. Nevertheless, a proportion of patients will relapse, and their prognosis is dismal. Advances in pathogenic mechanisms brought us a window of opportunity, but we need a great international, multicenter effort to design approaches capable of modifying the future.

## Author contributions

RC, JP, LL, and OB reviewed the literature and organized and wrote the article. All authors contributed to the article and approved the submitted version.
